# BH3-only proteins Puma and Beclin1 regulate autophagic death in neurons in response to Amyloid-β

**DOI:** 10.1038/s41420-021-00748-x

**Published:** 2021-11-15

**Authors:** Akash Saha, Suraiya Saleem, Ramesh Kumar Paidi, Subhas C. Biswas

**Affiliations:** 1 Cell Biology and Physiology Division, CSIR-Indian Institute of Chemical Biology, 4 Raja S. C. Mullick Road, Kolkata, 700 032 India; 2 Present Address: Suraiya Saleem, Bhupat and Jyoti Mehta School of Biosciences, Indian Institute of Technology Madras, IIT P.O., Chennai, 600 036 India; 3 Present Address: Department of Neurological Sciences, RUSH University Medical Center, Chicago, IL 60612 USA

**Keywords:** Cell death in the nervous system, Alzheimer's disease

## Abstract

Alzheimer’s disease (AD) is characterized by accumulation of senile amyloid-β (Aβ) plaques and hyperphosphorylated tau tangles causing progressive loss of synapse and neuronal death. Out of the various neuron death modalities, autophagy and apoptosis are reported to be the major death paradigms in AD. However, how these two processes lead to neuronal loss is still inconspicuous. Here we report that under Aβ toxicity, aberrant autophagy is induced with inefficient autophagic flux in neurons. Simultaneous activation of both autophagy and apoptosis are seen in primary cortical neurons as well as in transgenic mice brains. We found that induction of autophagy by rapamycin is detrimental for neurons; whereas downregulation of Beclin1, an important autophagy inducing protein, provides significant protection in Aβ treated neuronal cells by blocking cytochrome-c release from the mitochondria. We further report that downregulation of Puma, a BH3-only pro-apoptotic protein, inhibits the induction of aberrant autophagy and also ameliorates the autophagy flux under the influence of Aβ. Notably, stereotactic administration of shRNAs against Puma and Beclin1 in adult Aβ-infused rat brains inhibits both apoptotic and autophagic pathways. The regulation of both of the death processes is brought about by the direct interaction between Puma and Beclin1 upon Aβ treatment. We conclude that both Beclin1 and Puma play essential roles in the neuronal death caused by the induction of aberrant autophagy in AD and targeting their interaction could be vital to understand the crosstalk of autophagy and apoptosis as well as to develop a potential therapeutic strategy in AD.

## Introduction

The underlying pathogenesis of Alzheimer’s disease (AD) is selective synaptic loss followed by the death of neurons leading to memory impairment and dementia. The most accepted hypothesis on disease pathogenesis is the amyloid cascade hypothesis which suggests that the earliest pathological hallmark of the disease is the deposition of amyloid-β (Aβ) plaques that subsequently leads to tau deposition, neuronal loss, synaptic anomalies, and degeneration [1, 2]. This hypothesis is extended by Bart de Strooper and Eric Karran [3] and they proposed that the accumulation of plaques and tau tangle is a slow and gradual process that acts as a risk factor. The clinical manifestation of AD only occurs when there is a failure in the cellular homeostasis and is denoted by dysfunctional neurovascular unit, anomalous neuronal circuitry, aberrant microglial and astrocyte functions which altogether lead to neurodegeneration [4]. The two vital cell death mechanisms that influence neurodegeneration are apoptosis and autophagy [5–7].

Autophagy is a major intracellular catabolic pathway that eliminates damaged cellular contents like proteins, lipids, and other organelles, with the help of lysosomal enzymes [8–10]. Neurons are highly dependent on autophagy for their survival due to their extreme polarized and post-mitotic nature [11]. Autophagy maintains the health of neurons by preventing the accumulation of aggregated cytosolic wastes of proteins or membranes [12]. Accumulation of cellular wastes in neurons may incur a great burden in the cells which are not diluted due to their non-dividing nature [13, 14]. Knockout of *Atg* genes such as ATG5 [15] or ATG7 [16] are shown to be embryonically lethal and displayed behavioral anomaly in mice [17]. Genome-wide sequencing showed that a few genes like ATG5, ATG7, ATG101, and ATG16L1 are indispensable for neuronal survival [18]. However, the role of autophagy in AD is quite controversial. Accumulation of autophagy vacuoles (AVs) is reported in AD patients’ brains as compared to normal brain biopsies [19]. While few reports suggest autophagy being crucial in the degradation of Aβ plaques, APP [20–24] and tau tangles [25–27], there are many reports suggesting the presence of aberrant AVs containing Aβ aggregates in AD brains [28–31]. Since there are varied reports suggesting the beneficial and deteriorating effects of autophagy in AD, its exact role in AD pathogenesis still remains uncertain.

In AD, it is reported that the Aβ plaque may generate oxidative stress or trigger expression/activation of a number of pro-apoptotic proteins in neurons leading to cell death by apoptosis [32–36]. Reports of increased DNA fragmentation, granulated chromatin, changes in cell shape and size, caspase activity, changes in levels of the apoptosis-related proteins of the Bcl-2 family have been cited in the AD patients’ brains bearing senile plaque depositions [37, 38]. Bcl-2 protein family play important roles in the interplay of autophagy and apoptosis [39, 40]. Reports suggest that BH3-domain only pro-apoptotic protein, Bim, sequesters Beclin1 or ATG6, which is a vital autophagic protein and inhibits autophagic initiation in starved HeLa cells [41]. Puma (p53 upregulated modulator of apoptosis) is a potent pro-apoptotic protein like Bim; however, its role in autophagy is not yet revealed. Bcl-2 is supposed to have a dual characteristic; it is an anti-apoptotic protein but can also act as an anti-autophagic protein when it inhibits Beclin1 from associating with the autophagic machinery [39, 42]. Another level of crosstalk is served by the caspases. Wirawan and group identified two caspase-3 cleavage sites on Beclin1 [43] and the cleaved part is shown to sensitize the mitochondria for apoptosis. The C-terminal fragment of Beclin1 is reported to translocate to the mitochondria thus releasing apoptotic signals [44]. There are many levels of regulations and interactions between the autophagic and apoptotic machineries which are not yet clear. In this study, we concentrate primarily on the activation of both autophagy and apoptosis in neurons under the influence of Aβ and find out the role of autophagy in determining the neuronal fate. We specifically look into the effect of BH3-only proteins, Puma and Beclin1 in regulating autophagic death of neurons under Aβ toxicity.

## Results

### Aβ induces both autophagy and apoptosis

**Fig. 1:**
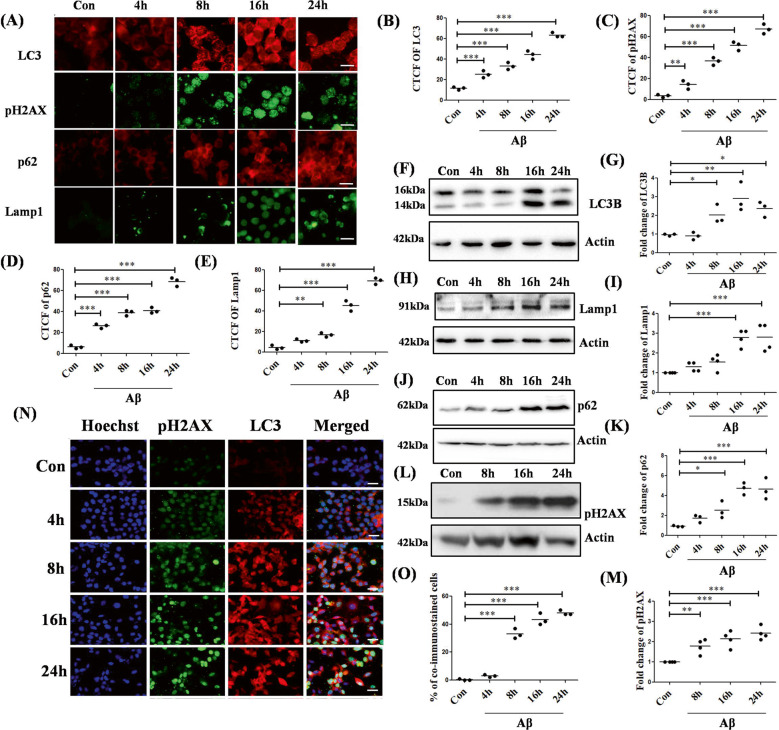
Aβ treatment induces both autophagy nad apoptosis in differentiated PC12 cells. (**A**) PC12 cells were primed and treated with 5 µM Aβ and stained with autophagy markers: LC3 (red), p62 (red), and Lamp1 (green) and apoptotic marker: pH2AX (green) in separate sets of experiments. Scale bars: 50 µm. (**B**), (**C**), (**D**), and (**E**) represents the corrected total cell fluorescence of LC3, pH2AX, p62, and Lamp1 respectively. Data represented were collected from 3 independent experiments. PC12 cells were primed and treated with 5 µM Aβ. Cell lystaes were used to asses the endogenous levels of (**F**) LC3B, (**H**) Lamp1, (**J**) p62, and (**L**) pH2AX respectively for the time points as indicated. (**G**), (**I**), (**K**), and (**M**) are the graphical representation of the fold change of the protein levels of LC3B, Lamp1, p62, and pH2AX respectively under Aβ toxicity. Data represented were collected from 3 independent experiments, except for (**I**) and (**M**) where data represented was from 4 independent experiments. (**N**) Primed PC12 cells were treated with 5 µM of Aβ and were stained with anti-LC3B antibody (red) and anti-pH2AX antibody (green) for co-immunocytochemistry studies. Nuclei were stained with Hoechst (blue). Scale bar 50 µm. (**O**) Graphical representation of the percentage co-immunostained cells under Aβ toxicity. Data represented were collected from 3 independent experiments. The asterisks denote statistically significant differences from control at corresponding time points:*p < 0.05, **p < 0.001, ***p < 0.0001.

We began our quest using neuronally differentiated PC12 cells being treated with oligomeric Aβ at a concentration of 5 µM for overnight (Supplementary Data, Fig. S1A–B). We checked the onset and progression of autophagy and apoptosis by checking the levels of their markers by immunocytochemistry. We found an increase in the expression of autophagosome markers such as LC3, p62, a lysosomal marker, Lamp1, and a marker of DNA double-strand breaks, pH2AX in differentiated PC12 cells under Aβ toxicity over a period of 0-24 h (Fig. 1A–E). The expression of these proteins gradually increased over time. We also checked for apoptosis by TUNEL assay under the same condition and obtained similar results (Supplementary Data, Fig. S2A–B). This upregulation of autophagic and apoptotic proteins were further checked by western blotting and an increase of LC3B, p62, Lamp1, and pH2AX levels was found as early as 8 h with a peak at 16 h (LC3B and p62) or 24 h (Lamp1 and pH2AX) post Aβ treatment (Fig. 1F–M). Having observed induction of both autophagy and apoptosis after Aβ treatment, we were intrigued to investigate the specific cellular population in which both these phenomena occurred simultaneously. Our co-immunocytochemistry studies revealed that following the Aβ insult, there was a gradual increase of cells exhibiting both LC3 and pH2AX, and the number of cells showing co-staining increased significantly in a time-dependent manner from 8 h to 24 h of treatment (Fig. 1N, O).

**Fig. 2:**
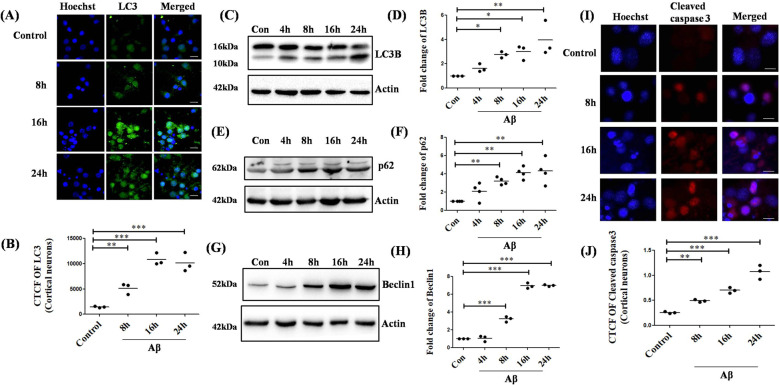
Aβ evokes autophagy and apoptosis in primary cortical neurons. (**A**) Primary rat cortical neurons (7DIV) were treated with 1.5 µM Aβ for indicated time periods and stained with an anti-LC3B antibody (green) to assess the endogenous levels of the protein. Nuclei were stained using Hoechst (blue). Scale bars: 50 µm. (**B**) Graphical representation of the corrected total cell fluorescence of LC3B. Data represented were collected from 3 independent experiments. Primary rat cortical neurons (7DIV) were treated with 1.5 µM Aβ for indicated time periods and the cell lysates were subjected to western blot analysis to check the levels of (**C**) LC3B, (**E**) p62, and (**G**) Beclin1 respectively. (**D**), (**F**) and (**H**) are the graphical representations of the fold change of the protein levels of LC3B, p62, and Beclin1 respectively. Data represented were collected from 3 independent experiments, except for (**F**) where data represented was from 4 independent experiments. (**I**) Primary rat cortical neurons (7DIV) were treated with 1.5 µM Aβ for indicated time periods and stained with an anti-cleaved caspase-3 antibody (red) to assess the endogenous levels of cleaved caspase-3. Nuclei were stained using Hoechst (blue). Scale bars: 50 µm. (**J**) Graphical representation of the corrected total cell fluorescence of cleaved caspase-3. Data represented were collected from 3 independent experiments. The asterisks denote statistically significant differences from control at corresponding time points; *p < 0.05, **p < 0.001, ***p < 0.0001.

Since PC12 cells is an immortal cell line, we checked the status of autophagic and apoptotic proteins in primary cortical neurons. We found a significant and gradual increase in the expressions of autophagic initiation marker, LC3B under Aβ insult in a time-dependent manner as checked by both immunocytochemistry and western blotting (Fig. 2A–D). Protein levels of another important autophagic protein, Beclin1, and autophagy flux marker, p62 were also elevated gradually and significantly under Aβ toxicity in cortical neurons from 8 h to 24 h (Fig. 2E–H). The apoptotic effector, cleaved caspase-3 staining was also shown to be increased under the influence of Aβ from 8 h to 24 h (Fig. 2I, J). Collectively, these data indicate that both apoptosis and autophagy are induced upon Aβ insult in neuronal cells and both phenomena occur simultaneously in a particular cell.

### Induction of autophagy does not protect cells from Aβ toxicity

**Fig. 3:**
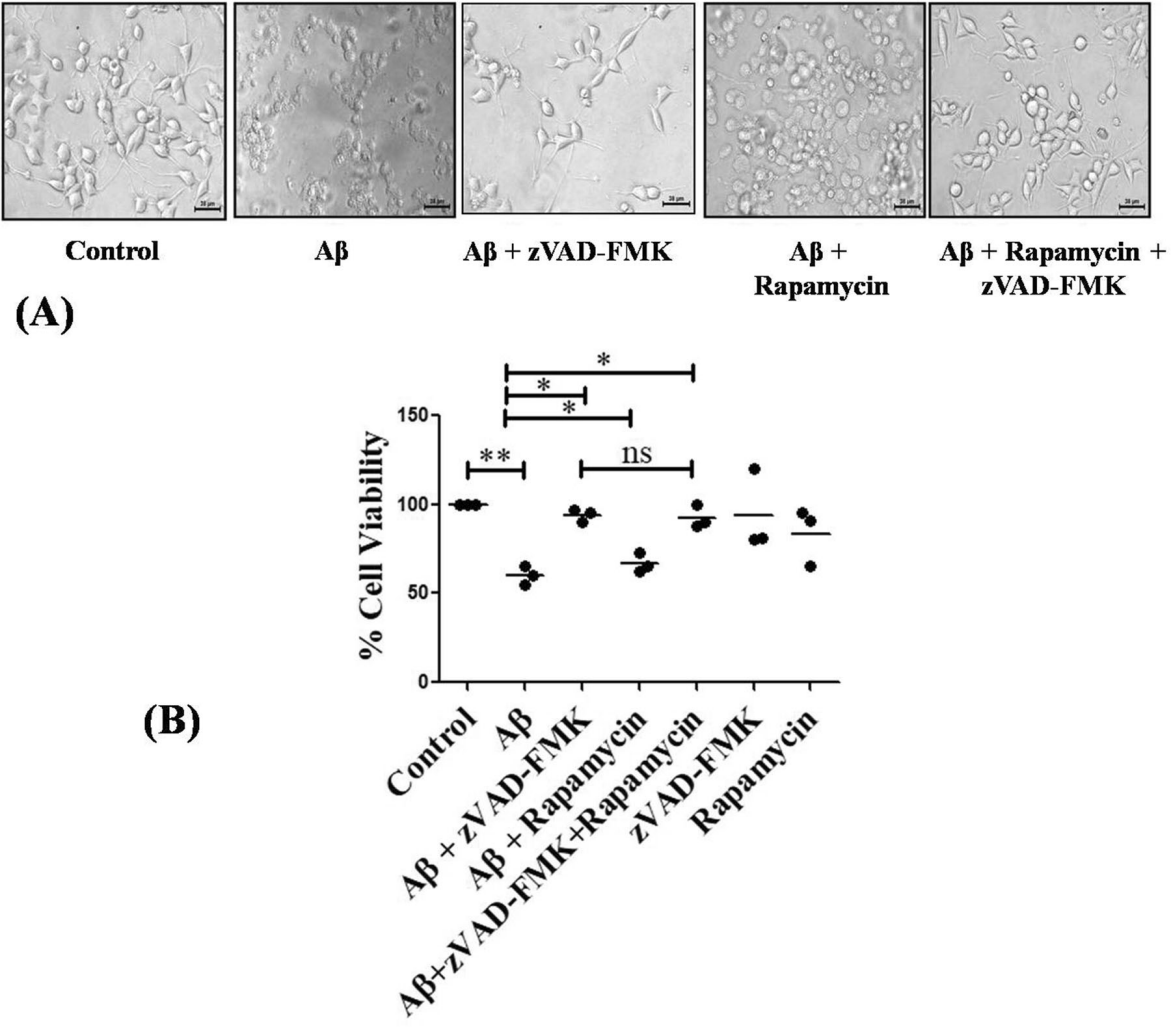
Activation of autophagy does not provide protection against Aβ treatment. (**A**) PC12 cells were primed and treated with 5 µM Aβ in the presence of autophagy inducer rapamycin; pan-caspase inhibitor zVAD-FMK and in conjunction of rapamycin and zVAD-FMK for 16 h and phase-contrast micrographs were taken. Scale bars: 38 µm. (**B**) Graphical representation of cell survival following Aβ treatment with zVAD-FMK, rapamycin, or both, for 16 h. Data represented were collected from 3 independent experiments. The asterisks denote statistically significant differences from control at corresponding conditions; *p < 0.05, **p < 0.001, ns represents no significance.

Since we found that both autophagy and apoptosis are induced in response to Aβ treatment in neuronal cells, we therefore intended to check if autophagy is beneficial on cell viability in presence of Aβ. We treated neuronally differentiated PC12 cells with Aβ for overnight in the presence of pan-caspase inhibitor (zVAD-FMK) to inhibit apoptosis, rapamycin to induce autophagy, or both zVAD-FMK (to inhibit apoptosis) and rapamycin (to simultaneously induce autophagy). Results showed that the death caused by Aβ was rescued when apoptosis was inhibited by zVAD-FMK. However, there was no rescue seen when autophagy was induced using rapamycin. Further, simultaneous induction of autophagy and inhibition of apoptosis by rapamycin and zVAD-FMK respectively did not provide any more significant protection as compared to when only apoptosis was blocked, suggesting that induction of autophagy does not play a major role in cell survival under Aβ insult, at least in cultured neurons (Fig. 3A–B).

### Beclin1 promotes neuronal cell death upon Aβ treatment

**Fig. 4:**
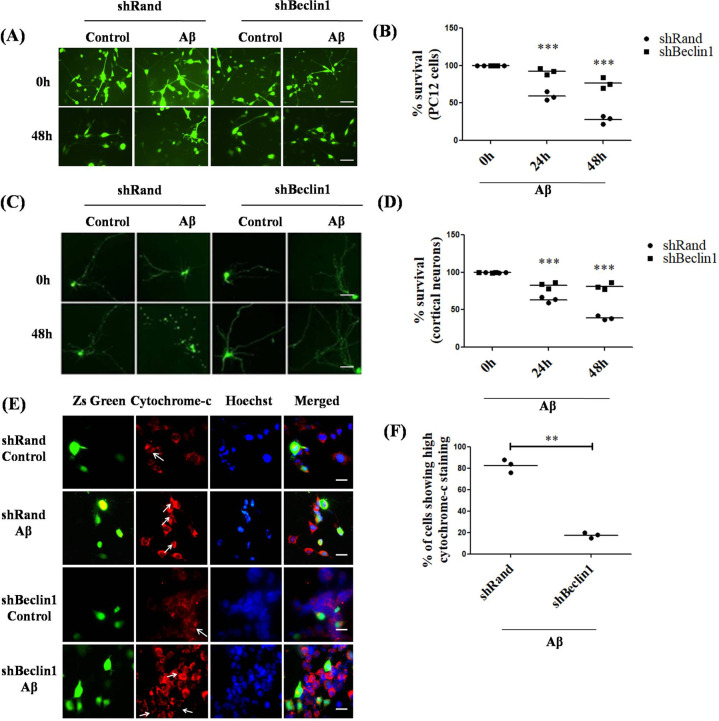
Downregulating Beclin1 protects neuronal cells against Aβ treatment. (**A**) Neuronally differentiated PC12 cells were transfected with shBeclin1-ZsGreen or shRand-ZsGreen. 48 h post transfection, cells were treated with 5 µM Aβ. Representative images of transfected cells that were maintained in the presence or absence of Aβ for indicated time periods are shown. Scale bars: 50 µm. (**B**) Graphical representation of the percentage of viable cells where live green cells were counted just after treatment and after 48 h of treatment with Aβ under a fluorescence microscope. Data represented were collected from 3 independent experiments. (**C**) Primary cultured rat cortical neurons (3 DIV) were transfected with shBeclin1-ZsGreen or control shRand-ZsGreen and maintained for 48 h and then subjected to 1.5 µM Aβ for 48 h. Representative pictures of transfected neurons that were maintained in the presence or absence of Aβ for indicated time periods are shown. Images were taken using an inverted fluorescence microscope. Scale bars: 50 µm. (**D**) Graphical representation of the percentage of viable green cells after each time point. The numbers of surviving transfected (green) cells were counted under a fluorescence microscope just after treatment and after 48 h of treatment with Aβ. Data represented were collected from 3 independent experiments. (**E**) Neuronal PC12 cells were transfected with shBeclin1-ZsGreen or shRand-ZsGreen. 48 h post-transfection, cells were treated with Aβ and immunostained for cytochrome-c and checked under a fluorescence microscope. Scale bars: 50 µm. (**F**) Graphical representation of the percentage of cells with high cytochrome-c staining after Aβ treatment in Beclin1 downregulated conditions. Data represented were collected from 3 independent experiments. The asterisks denote statistically significant differences from control (shRand) at corresponding time points or conditions; **p < 0.001, ***p < 0.0001.

Beclin1 is a major autophagy protein that has been reported to be induced under neurodegenerative conditions [53, 54] and plays an essential role in autophagy-apoptosis crosstalk [55]. Interestingly, recent evidences have also shown that proteolytic cleavage of Beclin1 causes apoptotic neuronal loss in two different models of neurodegeneration [56]. Since our previous experiments showed that autophagy induction does not protect neurons from Aβ toxicity, we next checked whether inhibition of autophagy by downregulating Beclin1 could provide protection to neurons. Neuronally differentiated PC12 cells were transfected with either shBeclin1 or shRand (control), and then were subjected to Aβ treatment (Fig. 4A, B) for 24 h and 48 h. Results showed significant protection, where Beclin1 was downregulated compared to cells transfected with shRand. Similar protection was obtained in cortical neurons treated with 1.5 µM Aβ upto 48 h (Fig. 4C, D). Knockdown of Beclin1 not only protects neuronal cells from death but also retains the overall neuronal morphology of neurons even after 48 h of Aβ treatment (Fig. 4C). We further observed that number of cells in which Beclin1 was downregulated, levels of cytochrome-c were significantly low as opposed to control plasmid transfected cells upon treatment with Aβ (Fig. 4E, F). Taken together, these results indicate that Beclin1 plays an essential role in neuron death evoked by Aβ by inducing both autophagy and apoptosis.

### Puma and FoxO3a evoke autophagy initiation but hinder autophagy flux in neuronal cells upon Aβ insult

**Fig. 5:**
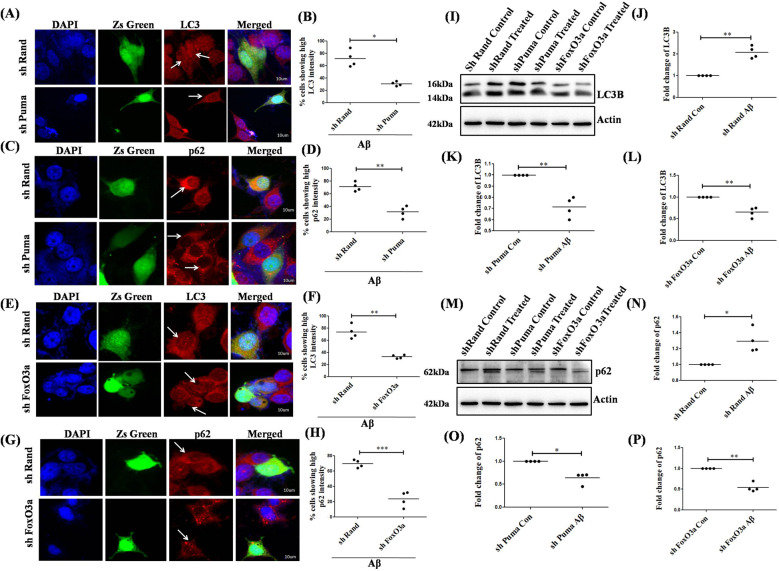
Puma and FoxO3a trigger autophagy initiation but interferes in autophagy flux upon Aβ toxicity. Naïve PC12 cells were transfected with shPuma-ZsGreen (green) or shRand-ZsGreen (green), primed, and then treated with 5 µM Aβ for overnight after which they were immunostained with (**A**) anti-LC3B antibody (red) and (**C**) anti-p62 antibody (red). Nuclei were stained with DAPI. Images were taken under a confocal microscope. Scale bars: 10 µm. (**B**) and (**D**) Graphical representation of the percentage of cells showing the high intensity of LC3B and p62 respectively under Puma compromised conditions. Data represented were collected from of 4 independent experiments. Naïve PC12 cells were transfected with shFoxO3a-ZsGreen (green) or shRand-ZsGreen (green), primed, and then treated with 5 µM Aβ for overnight after which they were immunostained with (**E**) anti-LC3B antibody (red) and (**G**) anti-p62 antibody (red). Nuclei were stained with DAPI. Images were taken under a confocal microscope. Scale bars: 10 µm. (**F**) and (**H**) Graphical representation of the percentage of cells showing the high intensity of LC3B and p62 respectively under FoxO3a compromised conditions. Data represented were collected from 4 independent experiments. Naïve PC12 cells were transfected with shPuma or shFoxO3a or shRand, primed, and then treated with 5 µM Aβ for overnight, and whole-cell lysates were assessed by western blot for protein levels of (**I**) LC3B and (**M**) p62 respectively. (**J**), (**K**) and (**L**) Graphical representation of the fold change of LC3B under Aβ toxicity in control, Puma downregulation, and FoxO3a downregulation respectively. Data represented were collected from 4 independent experiments. (**N**), (**O**), (**P**) Graphical representation of the fold change of p62 under Aβ toxicity in control, Puma downregulation, and FoxO3a downregulation respectively. Data represented were collected from 4 independent experiments. The asterisks denote statistically significant differences from control (shRand) at corresponding conditions; *p < 0.05, **p < 0.001, ***p < 0.0001.

We have previously shown the activation of Puma in response to Aβ in neurons leads to neuronal death [34] and it has been also shown that Puma plays an essential role in the regulation of mitophagy in cancerous cells [57]. Taking a cue from these studies, we checked the effect of downregulation of Puma, on the regulation of autophagy initiation and flux under Aβ toxicity. We observed a significant drop in the levels of LC3B (Fig. 5A, B) and p62 (Fig. 5C, D) in the differentiated PC12 cells in which Puma had been downregulated as compared to the control cells, suggesting that downregulation of Puma inhibited the autophagy initiation but helped in maintaining the autophagic flux, as indicated by the reduced levels of p62, which is otherwise seen to be accumulated and upregulated under the influence of Aβ. Since Puma is directly activated by a transcription factor FoxO3a [33, 34] and FoxO3a is known to play a major role in autophagy induction in cardiomyocytes for normal cardiac functioning [58], we checked if this transcription factor plays any role in autophagy initiation and maintaining the autophagy. Performing an immunocytochemistry analysis in FoxO3a downregulated PC12 cells, we found a significant decrease in autophagic initiation (Fig. 5E, F) and p62 accumulation (Fig. 5G, H) as compared to the control cells under Aβ toxicity. We also determined these protein levels by western blotting and found a decrease in the levels of LC3B (Fig. 5I–L) and p62 (Fig. 5M–P) in both shPuma and shFoxO3a transfected PC12 cells as compared to control transfected cells under Aβ treatment, indicating a dual role of both Puma and FoxO3a in the regulation of autophagy and apoptosis.

### Autophagy and Apoptosis is induced simultaneously in brains of 5xFAD mice

**Fig. 6:**
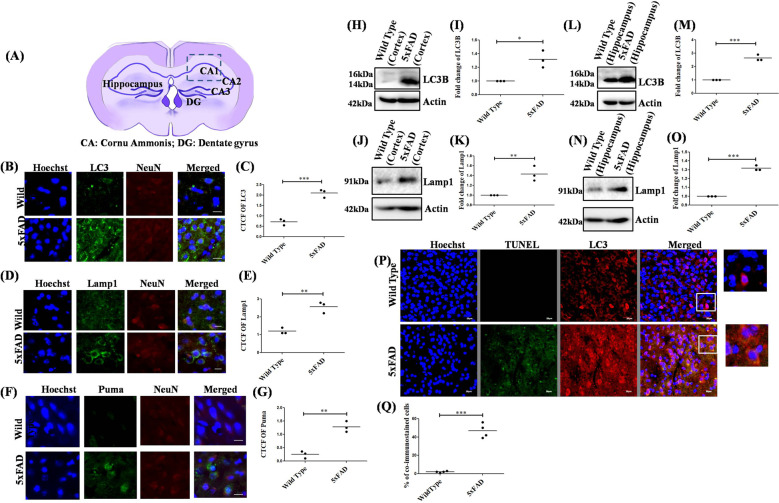
Apoptosis and autophagy are induced in 5XFAD mice brains. (**A**) Schematic representation of the brain section depicting the Cornu Ammonis 1 (CA1) region from where the microscopic images were collected. Brain sections (20 micron) from the CA1 region of 6 months old 5xFAD mice and wild type mice were taken and stained with Hoechst (blue) for nuclei, neuronal marker NeuN (red) and (**B**) anti-LC3B antibody (green) (**D**) anti-Lamp1 antibody (green) (**F**) anti-Puma antibody (green) respectively. Scale bars: 38 µm. (**C**), (**E**), and (**G**) Graphical representation of the corrected total cell fluorescence of LC3B, Lamp1, and Puma, respectively. *n* = 3 mice per group. Data represented were collected from 3 independent experiments. (**H**) and (**J**) represent western blot analysis of change in protein levels of LC3B and Lamp1 respectively of tissue lysates from the cortical region of 5xFAD mice brains as compared to the age-matched wild type mice. (**I**) and (**K**) Graphical representations of the fold change in autophagy proteins LC3B and Lamp1 in the cortical tissues of 5xFAD mice as compared to the wild-type mice. *n* = 3 mice per group. Data represented were collected from 3 independent experiments. (**L**) and (**N**) represent western blot analysis of change in protein levels of LC3B and Lamp1, respectively of tissue lysates from the hippocampal region of 5xFAD mice brains as compared to the age-matched wild type mice. (**M**) and (**O**) Graphical representations of the fold change in autophagy proteins LC3B and Lamp1 in the hippocampal tissues of 5xFAD mice as compared to the wild-type mice. *n* = 3 mice per group. Data represented were collected from 3 independent experiments. (**P**) Brain sections (20 micron) of 6 months5xFADand age-matched wild-type mice were immunostained with anti-LC3B antibody (red) and subjected to TUNEL assay (green). Scale bars: 20 µm. (**Q**) Graphical representation of the percentage co-stained cells in 5xFAD mice brains as compared to wild-type mice. *n* = 4 mice per group. Data represented were collected from 4 independent experiments. The asterisks denote statistically significant differences from wild type to transgenic; *p < 0.05, **p < 0.001, ***p < 0.0001.

To validate our in vitro studies on the occurrence of autophagy and apoptosis under Aβ toxicity, we first checked the autophagy levels in vivo in 12 months APP/PS1 transgenic mice brain and found an increase in the levels of both LC3 and p62 as compared to the wild type control mice brain (Supplementary Data, Fig. S3A–D). Next, we checked for the advent of autophagy and apoptosis in 6 months old 5xFAD mice brain as compared to the age-matched wild-type mice brain. Our immunohistochemistry studies show that there is an increase in the expression of autophagy marker proteins LC3 (Fig. 6B, C) and Lamp1 (Fig. 6D, E), in the 5xFAD mice brain compared to wild type. Also, we found an increase in the staining of Puma in the 5xFAD brain as compared to wild-type brains (Fig. 6F, G). Further, protein levels of autophagy markers were checked by western blot and we found significantly elevated levels of both LC3B and Lamp1 in 6 months old 5xFAD mice brain tissue lysates from the cortex (Fig. 6H–K) and hippocampus (Fig. 6L, O) as compared to the age-matched wild type mice cortex and hippocampus lysates respectively. In order to check if both the processes, viz, autophagy and apoptosis occurred simultaneously in the same cells, we performed the immunohistochemistry and TUNEL assays together in the same brain sections of 5xFAD mice and in corresponding wild type sections. We found an increase in the levels of LC3 and TUNEL positive cells in 5xFAD mice as compared to the wild-type set. Interestingly we also observed that many cells underwent both autophagy and apoptosis simultaneously in 5xFAD (Fig. 6P, Q). We thereby confirm that both autophagy and apoptosis simultaneously occur in the brain of AD mice. The level of crosstalk between both these processes may finally determine the survival or death of neurons.

### Down regulation of Puma and Beclin1 leads to inhibition of both autophagy and apoptosis in vivo

**Fig. 7:**
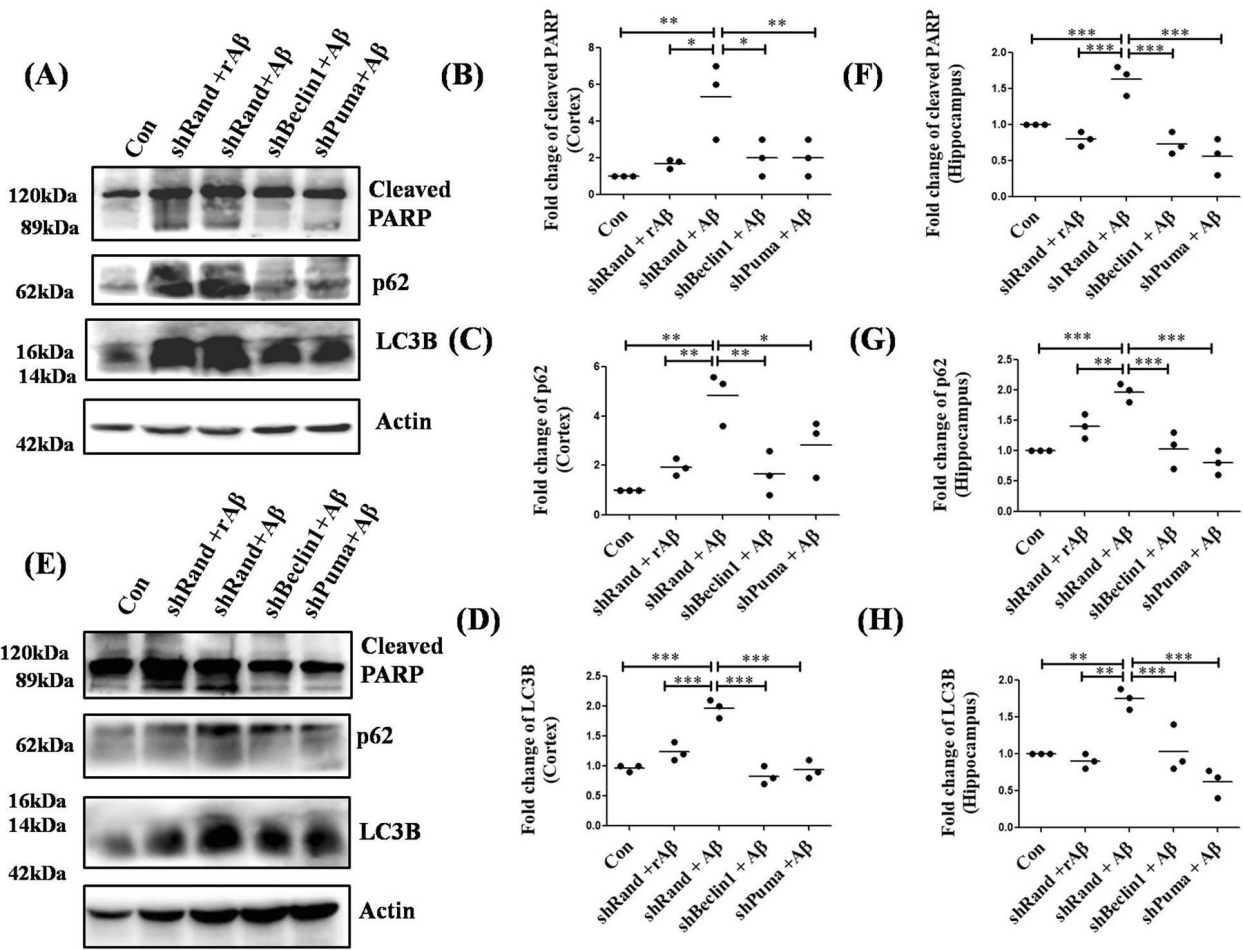
Down regulation of autophagy BH3-only protein Beclin1 and pro-apoptotic BH3-only protein Puma helps in cell survival and in autophagy flux maintenance. (**A**) Brain tissue lysates from the cortical area infused with Aβ along with shPuma or shBeclin1 were taken and assessed by western blot for LC3B, p62, and cleaved PARP. (**B**), (**C**) and (**D**) Graphical representations of the fold change of cleaved PARP, p62, and LC3B respectively in the cortical lysates of the animals. *n* = 3 rats per group. Data represented were collected from 3 independent experiments. (**E**) Brain tissue lysates from the hippocampal region were taken and assessed by western blot for LC3B, p62 cleaved PARP. (**F**), (**G**) and (**H**) Graphical representations of the fold change of cleaved PARP, p62, and LC3B respectively in the hippocampal lysates of the animals. *n* = 3 rats per group. Data represented were collected from 3 independent experiments. The asterisks denote statistically significant differences between corresponding conditions; *p < 0.05, **p < 0.001, ***p < 0.0001.

Since both autophagy and apoptosis are triggered simultaneously in both cellular and animal models of AD, we next checked the role of two key BH3-only proteins, Beclin1 (essential for autophagosome formation) and Puma (a pro-apoptotic protein) in cell death in an in vivo model. We created an animal model of AD by injecting oligomeric Aβ in the cortex of rat brains by stereotaxy. Puma and Beclin1 were downregulated in Aβ-infused rats by injecting specific shRNAs against them, exclusively. We infused reverse Aβ peptide (rAβ) in rat brains, to create negative controls and we infused PBS in the rat brains for control, in similar locations. We took the tissue lysates of the cortex and hippocampus of the brains of these rats and performed western blotting where we found a significant reduction in the levels of autophagy proteins LC3B and p62 in the cortex (Fig. 7A–D) and hippocampus(Fig. 7E–H), in both Puma or Beclin1 knockdown rat brains as compared to the Aβ-infused rat brains. We also found a decrease in the cleaved PARP levels in the brains of rats where we downregulated Puma or Beclin1 as compared to that of the Aβ-infused rat brains (Fig. 7A, B, E, F), suggesting that both Puma and Beclin1 play an essential role in the regulation of both autophagy and apoptosis, in determining the fate of neurons in AD.

### Autophagic BH3-only protein Beclin1 interacts with pro-apoptotic BH3-only protein Puma

**Fig. 8:**
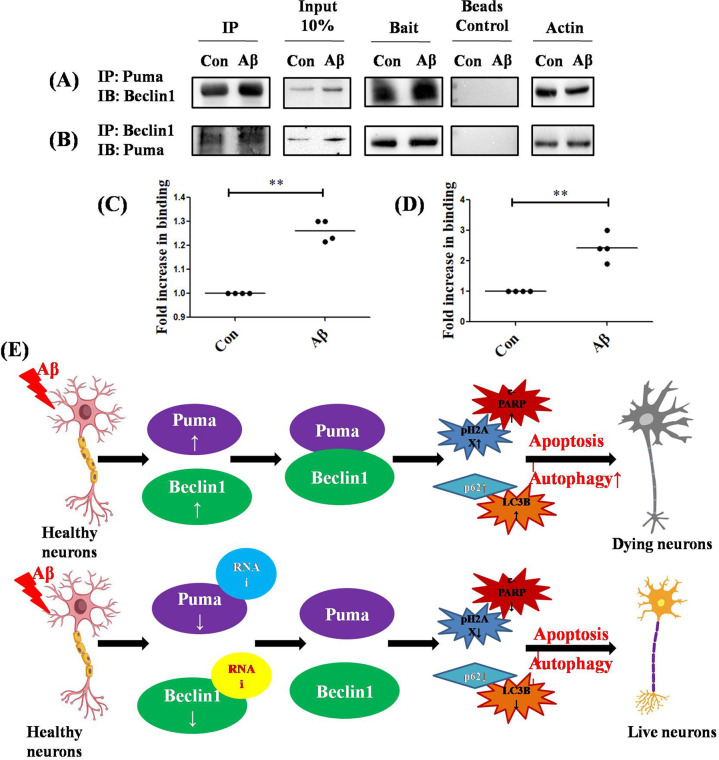
Autophagic BH3-only protein Beclin1 interacts with pro-apoptotic BH3-only protein Puma in cortical neurons. **A** Primary rat cortical neurons were cultured and treated with and without 1.5 µM Aβ for overnight and the cell lysates were immunoprecipitated (IP) with Puma antibody and immunoblotted (IB) for Beclin1. **B** Reverse immunoprecipitation was also performed to check the interaction of Beclin1 with Puma; cell lysates were immunoprecipitated with Beclin1 antibody and immunoblotted for Puma. The inputs and baits for the respective immunoprecipitation studies are shown in the second and the third column of (**A**) and (**B**). The beads controls are shown in the fourth column and the loading controls (Actin) for each immunoprecipitation study are represented in the fifth column of (**A**) and (**B**). (**C**) and (**D**) Graphical representation of the fold change in the interaction between Beclin1 and Puma under Aβ treatment as compared to control. Data represented were collected from 4 independent experiments. The asterisks denote statistically significant differences between corresponding conditions; **p < 0.001. **E** Schematic representation depicting the role of BH3-only proteins Puma and Beclin1 in the regulation of autophagic death in neurons exposed to Aβ.

Structural studies indicate that Beclin1 is a BH3-only protein with a BH3 binding motif through which it interacts with Bcl-2/Bcl-xL [59]. Reports have prompted towards a close interaction between BH3-only proteins Bim and Beclin1 which holds the essence of regulation of autophagy in starved conditions [41]. Taking a cue from such reports, we investigated whether Puma, due to the presence of BH3 motif, interacted with Beclin1 at any level in our model. Since we found that downregulation of Puma or Beclin1 leads to a substantial amount of inhibition of autophagy as well as apoptosis, it was imperative to have checked whether these proteins show any kind of interaction between them or not. We performed immunoprecipitation studies on tissue lysates of cortical neurons. Our study suggested a potential interaction between Puma and Beclin1. Furthermore, we see an increase in the interaction of the two BH3-only proteins under Aβ treated conditions as compared to the control (Fig. 8A–D). Collectively, these findings hint towards the fact that pro-apoptotic protein Puma regulates autophagy by its close association with Beclin1 which is an important autophagy protein (Fig. 8E). Downregulation of any of these proteins blocks initiation of autophagy and accumulation of cargo leading to the survival of neurons.

## Discussion

In this study, we investigated whether two BH3-only proteins - Beclin1, an inducer of autophagy, and Puma, a pro-apoptotic protein, interact with each other and influence neuron death in AD.A set of experimental findings suggest that both Beclin1 and Puma binds with each other and play necessary roles in autophagy induction but are responsible for impaired autophagy flux and apoptotic death of neurons. First, both autophagy and apoptosis are induced simultaneously in neurons upon Aβ exposure and in transgenic AD mice. Autophagolysosomes formation is induced as determined by the increase in levels of LC3B and Lamp1, however autophagy flux is reduced as p62 levels are increased. The induction of apoptosis is determined by the levels of pH2AX. Second, induction of autophagy is not beneficial for neurons as rapamycin does not block neuron death evoked by Aβ. However, inhibition of autophagy by downregulating Beclin1 protects neurons from death induced by Aβ. Third, Puma and its transcriptional regulator FoxO3a, play an essential role in autophagy induction and impaired autophagy flux. Fourth, both Beclin1 and Puma are responsible for the induction of autophagy and reduced autophagy flux in the Aβ-infused rat brain. Importantly, both are essential for PARP cleavage, an important marker of caspase-dependent apoptosis. Finally, Beclin1 and Puma directly interact with each other and this interaction is increased in response to Aβ treatment.

Besides Beclin1, Atg5 is reported to play a major role in the autophagy-apoptosis crosstalk. It is reported that Atg5 can trigger autophagic cell death by interacting with FADD which promotes apoptosis [60]. Evidences show that Atg5 is cleaved by calpains and the N-terminal fragment reaches the mitochondria and induces apoptosis by releasing cytochrome-c and activation of caspases [61]. Puma has shown to be regulated by autophagy in Tumor Necrosis factor-related Apoptosis-Inducing Ligand (TRAIL) mediated cancer cell death [62]. Alternatively, Puma plays a necessary role in protein clearance in neurons in response to pesticide, chlorpyrifos [63]. Puma has also been shown to play an essential role in mitophagy in cancer cells [57]. Moreover, both autophagic and apoptotic features are seen in dying neurons in cases of neonatal hypoxia-ischemia [64]. Along with the occurrence of the aforesaid processes, we now show that both autophagy and apoptosis occur simultaneously in neurons, and Beclin1 and Puma play an essential role on the induction of autophagy and apoptosis in Aβ induced neurodegeneration.

Although we found an induction of autophagy in neuronal cells, the accumulation of autophagy residual aggregates, as marked by p62, takes place at very early stages upon Aβ toxicity. Nixon’s group noted the accumulation of subcellular aggregates and AVs in dystrophic neuritis [65] and further studies have supported the accumulation of these AVs associated with dying neuritis in APP/PS1 mice [66]. In other studies, Nixon’s group showed that apoptosis was the mode of neuronal death in aging APP/PS mice and was the source of crosstalk between apoptosis and autophagy [67]. Consistent with that, we also observed the increase in levels of autophagy in APP/PS1 mice brain. Additionally, we found simultaneous activation of both autophagy and apoptosis in 6 months old 5xFAD mice brain compared to the age-matched wild-type mice brains.

Further we observed that induction of autophagy by rapamycin, which inhibits mTOR – the master regulator of autophagy, provides no beneficial effect on the viability of cells under Aβ treatment; on the contrary, inhibiting autophagy with 3-Methyladenine provided some protection to neurons [68]. As expected, inhibiting apoptosis by zVAD-FMK significantly helped in cell viability. However, there was no synergistic effect seen in cell survivability under apoptosis inhibited-autophagy induced conditions compared to apoptosis inhibited conditions. To further establish our findings on the negative role of autophagy in neurodegeneration, we downregulated Beclin1 in neuronal cells and found that upon Aβ treatment the viability of the neurons is better in Beclin1 downregulated conditions as compared to the Aβ treated cells.

Interestingly, we found that knocking down of Puma in in vitro and in vivo significantly reduces the induction of autophagy but interestingly helps in maintaining the autophagic flux which is impaired in response to Aβ. This proves that proteins that are pro-apoptotic in nature or which help in directly activating the apoptotic process regulate autophagy in Aβ induced toxicity by affecting its induction and flux. Alternatively, when autophagy inducer Beclin1 was downregulated, apoptosis is inhibited after Aβ treatment. We could therefore infer that the inhibition of both the processes, viz, autophagy and apoptosis is beneficial for the neurons under Aβ toxicity and that Puma has dual effects on autophagy. It reduces the induction of autophagy and secondly it promotes the flux of the already accumulated autophagic cargo, which eventually helps in the survival of the cells. We speculate that this regulation is brought about by a close interaction between Puma and Beclin1. The previous report has suggested an interaction between Bim and Beclin1 under starved conditions [41]. Our study reveals a direct interaction between Puma and Beclin1 upon Aβ treatment. However, final outcome differs as Bim inhibits autophagy, whereas Puma induces autophagy but results in an impaired autophagic flux which causes neuron death. Further investigation is required to reveal this disparity between two similar molecules and to understand how Puma leads to impaired autophagic flux in AD.

## Materials and methods

### Materials

Lyophilized Aβ_1-42_ was purchased from AlexoTech AB (Umea, Sweden). 1,1,1,3,3,3 hexafluoro-2-propanol (HFIP), insulin, progesterone, transferrin, human recombinant nerve growth factor (NGF), poly-D-lysine, putrescine, and selenium were purchased from Sigma (St. Louis, MO, USA). Anti-cleaved PARP (#9542 T) and anti-pH2AX (#2577) antibodies were from Cell Signaling Technology (Denver, MA, USA). Anti-p62 (#MAB 8028) antibody was purchased from R&D Systems (Minneapolis, MN, USA) and anti-Lamp1 was from Abcam (Cambridge, UK). Anti-LC3 (#NB100-2220), anti-Beclin1 (#NB500-249), anti-Puma (NBP1-76639) and anti- cleaved caspase 3 (#NB100-56113) antibodies was purchased from Novus (Colorado, USA). Anti-actinβ (#A3854) was procured for Sigma Aldrich (St. Louis, MO, USA). Anti-NeuN (MAB 377) was purchased from Millipore (Burlington, MA, USA). Anti-MAP2 antibody (sc-74421), Protein A agarose were from Santa Cruz Biotechnology (Dallas, Texas, USA). HRP conjugated secondary antibodies (#7074 S and #7076 S) were procured from Cell Signaling Technology (Denver, MA, USA). 4’, 6-diamidino-2-phenylindole (DAPI), Lipofectamine 2000, Alexa Fluor 488 (#A11008 and #A11001), Alexa Fluor 546 (#A11010 and #A11003), culture media, and serum were purchased from Invitrogen, (Life technologies, Grand Island, NY, USA). Rapamycin (#55310) and zVAD-FMK (#627610) were procured from Calbiochem (CA, USA). Brain tissues of APPswe-PS1de9 (APP/PS1) mice and control littermates were kindly gifted by Dr. Anant B Patel [45].

### Cell culture

Cortical neurons were isolated from the neocortex of E18 day embryonic rat brains and were cultured and maintained on poly-D-lysine-coated culture plates in DMEM/F12 medium with glucose (6 mg/ml), progesterone (20 ng/ml), insulin (25 µg/ml), transferrin (100 µg/ml), putrescine (60 µg/ml) and selenium (30 ng/ml). The neurons were maintained for 6 days after which they were subjected to treatment [46, 47]. Rat pheochromocytoma (PC12) cells (Research Resource Identifier, RRID: CVCL_0481) were cultured and maintained in DMEM medium (with glucose) supplemented with 10% heat-inactivated horse serum (HS) and 5% heat-inactivated fetal bovine serum (FBS) [48]. Differentiation of PC12 cells were brought about by NGF (50 ng/ml) in a medium containing 1% horse serum for 5 days prior to treatment [49]. PC12 cells were authenticated using a neuron-specific marker (immunostained with anti-MAP2 antibody), before use and were maintained up to 30 passages. For primary and secondary cell lines, 3-4 independent cell samples were processed and all of them were used for experimental studies and analysis. For all experiments, the outcomes were crosschecked by a third person who was completely blinded towards the experimental conditions.

### Animal models

Adult male Sprague Dawley (SD) rats weighing around 300-380 g were used. The animals were housed a maximum of three per cage in a temperature-controlled room (24 ± 2 °C), with 12–12 h light-dark cycle, humidity (60 ± 5%) and allowed to food and water *ad libitum* in the animal house of CSIR-Indian Institute of Chemical Biology, Kolkata. Adult 5xFAD mice (Swedish, Florida, and London mutations in APP and the M146L and L286V mutations in PSEN1 and age-matched wild type control C57BL/6 mice were procured from Jackson’s Laboratory). The mice were housed a maximum of five mice per cage in a temperature-controlled room and 12–12 h light-dark cycle, humidity as mentioned above. A set of 3 animals per group were used in the experiments since we performed only biochemical analysis.

### Preparation of Aβ oligomers

Purified and lyophilized Aß_1-42_ powder was reconstituted in HFIP to a concentration of 1 mM. The reconstituted solution was subjected to Speed Vac for 30–35 min for the complete removal of HFIP by evaporation. The obtained pellet was then resuspended in anhydrous DMSO to a concentration of 5 mM followed by sonication in a water bath for 10 min at 37 °C. This stock solution was then stored at −80 °C. The stock solution was further processed and diluted to an intermittent concentration of 400μM using PBS and SDS was added to a final concentration of 0.2%. This solution was incubated at 37 °C for 18–24 h. The preparation was further diluted to a final concentration of 100μM using PBS and incubated at 37 °C for 18–24 h before use.

### Oligomeric Aβ treatment to cells

Freshly prepared oligomeric Aβ_1-42_ was added to the medium containing neuronal cells for the specific time points as required. For neuronally differentiated PC12 cells, the concentration of oligomeric Aβ_1-42_ used was 5 μM and for primary rat cortical neurons, the concentration used was 1.5 μM [33, 34].

### Western Blot analysis

Treated and untreated cortical neurons or neuronally differentiated PC12 cells were lysed and proteins were analyzed by western blotting [50]. 30-50 μg of protein for each condition was resolved in 8–12% SDS-PAGE as per the need followed by their transfer on to PVDF membrane (Hybond: GE Healthcare, Buckinghamshire, UK). The proteins were then probed with the desired primary antibodies at 4 °C for overnight. HRP-conjugated secondary antibodies against the primary antibodies were used. The blots were detected using Biorad western blotting detection reagent, according to the manufacturer’s protocol. Imaging of all Western blots was performed using an Azure Chemidoc system using manufacturer’s protocol.

### Immunocytochemical staining

Neuronally differentiated PC12 cells and/or primary cortical neurons were fixed with 4% paraformaldehyde for 10 min. The cells were washed thrice with PBS for 5 min each, thoroughly. The cells were then blocked in 3% goat serum in PBS containing 0.3% Triton-X 100 at room temperature for 2 h. The cells were then immunolabelled with the desired primary antibody in the blocking solution for overnight at 4°C. The next day, the cells were washed in PBS thrice for 5–10 min each, followed by incubation with the appropriate secondary antibody for 2 h at room temperature. Nuclei were stained with Hoechst or DAPI. High-resolution images were taken using Leica TCS SP8 microscope (Wetzlar, Germany). For co-immunocytochemistry, the cells were first incubated with TUNEL reagent as per manufacturer’s protocol followed by incubation with primary and secondary antibodies. Fluorescence intensities of staining for control or treated cells were quantified using NIH ImageJ software. The corrected total cell fluorescence (CTCF) was determined by considering the integrated density of staining, area of the cell, and the background fluorescence for the different experimental conditions. CTCF = Integrated density − (area of selected cell × mean fluorescence of background readings).

### Immunoprecipitation

Primary cultured cortical neurons were either treated with 1.5 µM Aβ for 16 h (overnight) or left as untreated controls and interactions of Beclin1 with Puma were detected by co-immunoprecipitation assays. The cells were washed with PBS thrice for 5 min each and lysed. For immunoprecipitation, the agarose-conjugated anti-Beclin1 antibody was prepared by incubating 3 μg of anti-Beclin1 antibody with 20 μl of protein A agarose beads at 4 °C for 2 h in a shaking condition. The agarose-conjugated Beclin1 antibody was then pooled down and incubated with treated and untreated cell lysates containing equal amounts of protein for overnight at 4 °C on a shaker. Following this incubation, antigen-antibody complexes were isolated and dissociated by boiling in sample buffer for 4 min. The agarose beads were centrifuged and the supernatant was subjected to western blot analysis as described previously, for the expression of Puma.

### Transfection

DNA was isolated using a Plasmid Maxi kit (Qiagen, Waltham, MA, USA). For survival assay, PC12 cells and/or primary cortical neurons were transfected with 0.5 μg of either pSIREN-Puma-shRNA-ZsGreen (shPuma) or pSIREN-FoxO3a-shRNA-ZsGreen (shFoxO3a)or pSIREN-Beclin1-shRNA-ZsGreen (shBeclin1) or pSIREN-Rand-shRNA-ZsGreen (shRand). Transfections were performed using lipofectamine 2000 in 500 μl of a serum-free medium in each well of a 24-well plate. Lipofectamine-containing medium was replaced by fresh medium after 4–6 h of transfection. Transfection was performed on the third day of culture for primary cortical neurons. Differentiated neuronal PC12 cells were transfected on the third day of differentiation. For endogenous Puma or Beclin1or FoxO3adownregulation, naive PC12 cells were transfected with either 1.5 μg of shPuma or shBeclin1 or shFoxO3a or shRand, followed by differentiation and treatment. Transfections were done in 1.5 ml of serum-free medium per well of a 6-well plate using lipofectamine 2000.

### Survival Assay

Primary cortical neurons (3DIV) and differentiated PC12 cells (3DIV) were transfected with shRand or shBeclin1 as mentioned above. The cells were exposed to oligomeric Aβ_1-42_after 48 h of transfection. The number of transfected neurons (green) was counted (0 h) under the microscope. The transfected and surviving neurons were again counted after 24 h and 48 h of treatment [49]. Control and Aβ-treated transfected neurons were imaged under Leica TCS SP8 microscope (Wetzlar, Germany).

### TUNEL assay

TUNEL positive cells in Aβ_1-42_ treated PC12 cells or in brain sections of transgenic mice models were detected using the in-situ cell death detection kit (Clonetech, CA, USA). The steps involved in the process were followed as given in the manufacturer’s protocol.

### Oligomeric Aβ infusion in animals

Male Sprague Dawley (SD) rats (300–380 g) were infused with oligomeric Aβ as described previously [51]. Rats were anaesthetized by injecting a mixture of xylazine–ketamine, placed on a stereotaxic frame. A volume of 5 μl of 100 µM Aβ in PBS was infused in the right cerebral cortex at stereotaxic co-ordinates from bregma: AP: −4.1, ML: 2.5, DV: 1.3 mm, according to the rat brain atlas and a previous report [51]. An equal volume of PBS was injected in control animals. 2 µg of pSIREN-Puma-shRNA-ZsGreen (shPuma) or pSIREN-Beclin1-shRNA-ZsGreen (shBeclin1) or pSIREN-Rand-shRNA-ZsGreen (shRand) was infused in the rat cortices along with the Aβ to create Puma-deficient, Beclin1-deficient and sham control animal models of AD [52]. Reverse amyloid-beta peptide, Aβ_42-1_ (rAβ) was also injected in similar loci of the rat brains to create a negative control model. Randomizations of the animals were done for the above groups such that similar age and weighted animals were selected for the stereotactic infusion. After infusion, every animal was considered for the experimentation. No animals were excluded for the present study. Animals have sacrificed 11 days post-injection. The brains were dissected out following cardiac perfusion and fixed in 4% PFA for 24 h and were then incubated in a 30% sucrose solution for 24 h and then cryo-sectioning was performed by using a cryotome (Thermo, West Palm Beach, FL, USA). Collection of tissue samples for western blot analysis and sectioning were done blindly. Tissue samples for western blot analysis were taken from the cortical and hippocampal regions of dissected and non-perfused brains and subjected to tissue homogenization and quantification.

### Immunohistochemistry of brain slices

Cryo sections measuring 20 µm of the brains from Aβ-infused or PBS-infused rats (as described earlier) or wild-type or APP/PS1 (Swedish mutation in APP and PS1 mutation) or 5xFAD transgenic mice were taken and blocked with 5% goat serum in PBS containing 0.3% Triton-X 100 for 1 h at room temperature. The sections were then incubated with the desired primary antibody in a blocking solution for overnight at 4 ˚C. The following day, the sections were washed with PBS thrice and then incubated with a fluorescence-tagged secondary antibody for 2 h at room temperature. The nuclei were stained with Hoechst or DAPI. The sections were mounted in anti-fade reagent and high-resolution images were taken using Leica TCS SP8 microscope. For co-immunohistochemistry, the sections were first incubated with TUNEL reagent as per manufacturer’s protocol followed by incubation with primary and secondary antibodies.

### Statistics

Experimental results are reported as the mean of three individual experiments using GraphPad Prism 5 software. Student’s t-test was performed as paired or unpaired wherever required, two-tailed sets of arrays to evaluate the significance of the difference between the means and is presented as p-values. For multiple conditions, one-way ANOVA was performed wherever required, to evaluate the significance of the difference between the means and are presented as p-values. Newman-Keuls Multiple Comparison post-test was performed. 95% confidence interval was considered for the significance. For survival assays, two-way ANOVA was performed and Bonferroni post-tests were performed to evaluate the variance and significance which is presented as *p*-values.

## Supplementary information

Supplementary material

## Data Availability

The datasets used and/or analyzed during the current study are available from the corresponding author on reasonable request.
