# Correction: BH3-only proteins Puma and Beclin1 regulate autophagic death in neurons in response to Amyloid-β

**DOI:** 10.1038/s41420-021-00772-x

**Published:** 2021-12-10

**Authors:** Akash Saha, Suraiya Saleem, Ramesh Kumar Paidi, Subhas C. Biswas

**Affiliations:** 1 Cell Biology and Physiology Division, CSIR-Indian Institute of Chemical Biology, 4 Raja S. C. Mullick Road, Kolkata, 700 032 India; 2 Present Address: Suraiya Saleem, Bhupat and Jyoti Mehta School of Biosciences, Indian Institute of Technology Madras, IIT P.O., Chennai, 600 036 India; 3 Present Address: Department of Neurological Sciences, RUSH University Medical Center, Chicago, IL 60612 USA

**Keywords:** Cell death in the nervous system, Alzheimer's disease

Correction to: *Cell Death Discovery* 10.1038/s41420-021-00748-x, published online 15 November 2021

The original version of this article unfortunately contained a mistake in an author name. The correct name is Ramesh Kumar Paidi. The original article has been corrected.

