# Supplementary material for: BH3-only proteins Puma and Beclin1 regulate autophagic death in neurons in response to Amyloid-β
Source: Cell Death Discov. 2021 Nov 15;7:356. doi: 10.1038/s41420-021-00748-x (PMC8593071; doi:10.1038/s41420-021-00748-x)
Supplement: Supplementary file 1 — Supplementary material [file 41420_2021_748_MOESM1_ESM.docx]

**Supplementary Material**

**Figure S1: Aβ causes death in differentiated PC12 cells.**

**
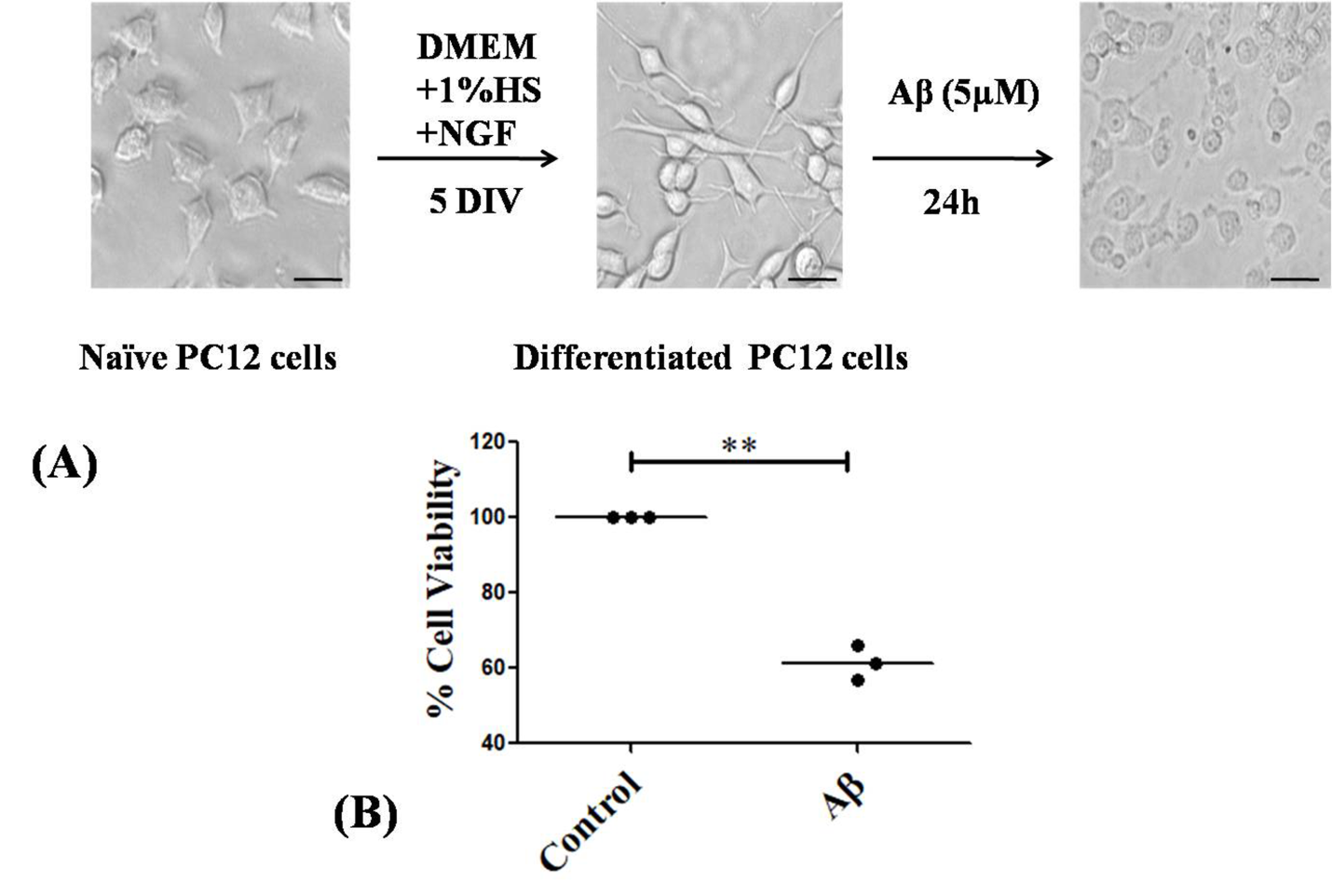
**

(A) Naïve PC12 cells were primed for 5 DIV followed by 5µM Aβ treatment for 24h. Scale bars: 38µm. Data represented was collected from 3 independent experiments. (B) Graphical representation of percentage of viable PC12 cells after 24h of Aβ treatment. Asterisks denote statistically significant differences; **p<0.001.

**Figure S2: Both autophagy and apoptosis are activated simultaneously in differentiated PC12 cells upon Aβ insult.**

**
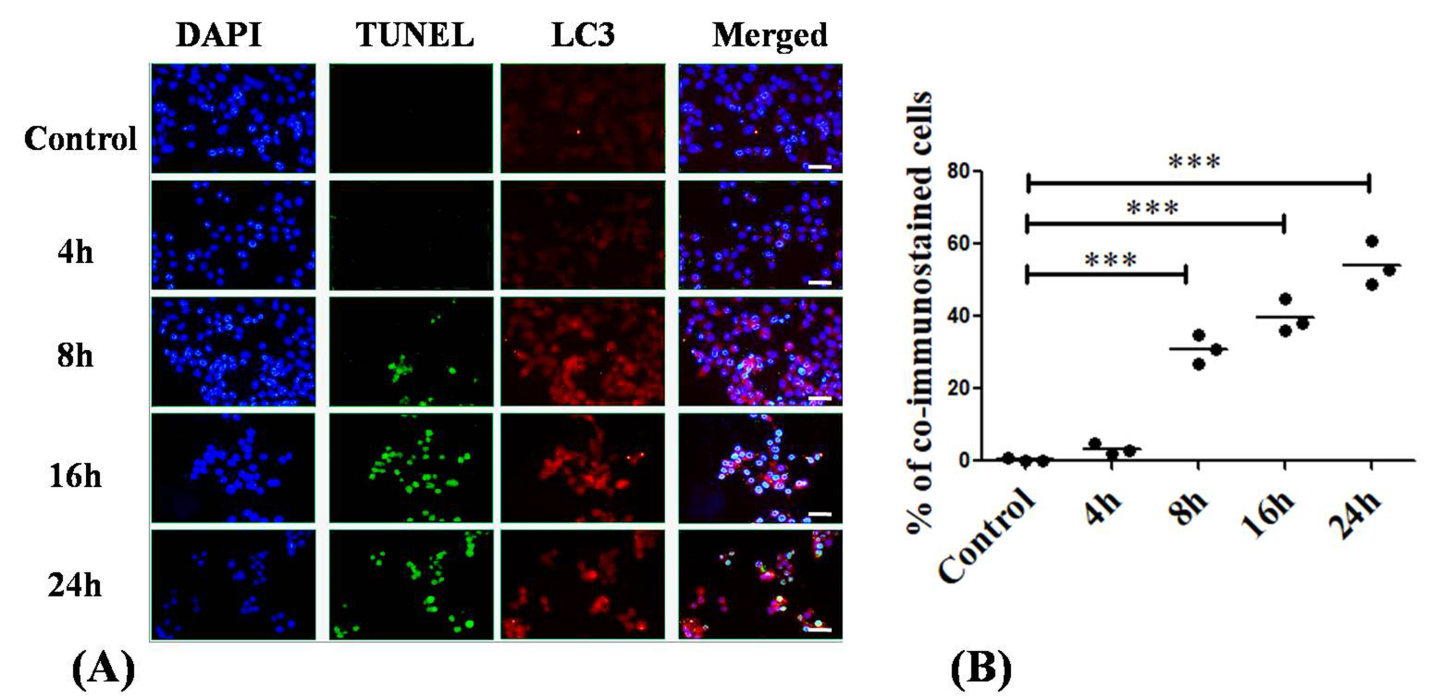
**

(A) Primed PC12 cells were treated with 5µM of Aβ and were subjected to TUNEL assay (green) followed by immunostaining for LC3. Scale bars: 50µm. Data represented was collected from 3 independent experiments. (B) Graphical representation of the percentage of cells co-immunostained. Asterisks denote statistically significant differences; ***p<0.0001.

**Figure S3: Impaired autophagy is triggered in APP/PS1 mice brains.**

**
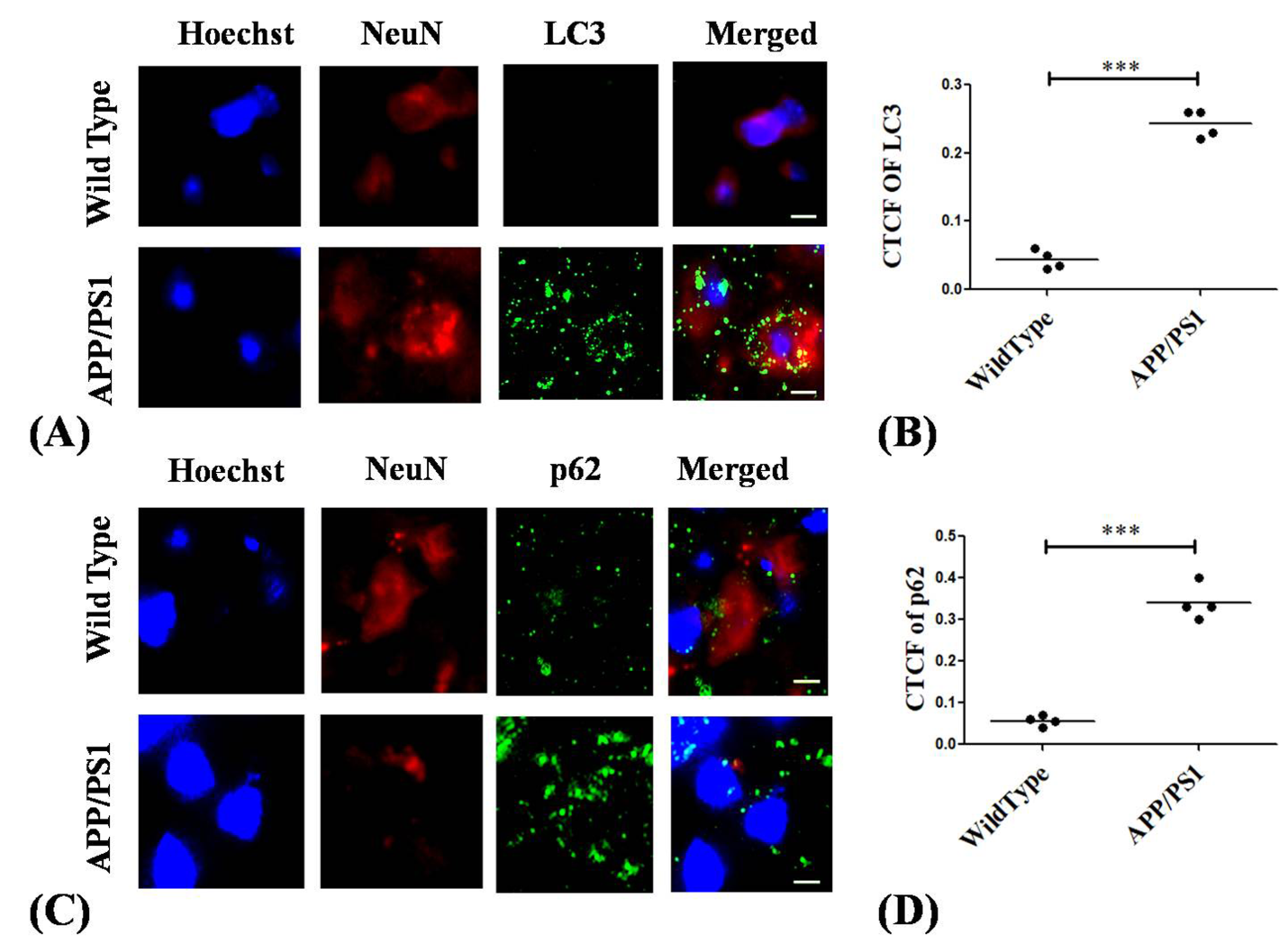
**

Brain sections (20µm) of 12 months old of APP/PS1 were taken and immunostained for (A) LC3 (B) p62. Scale bars: 38µm. Data represented was collected from 4 independent experiments. (C) and (D) Graphical representation of the total cell fluorescence of LC3 and p62 respectively. Asterisks denote statistically significant differences; ***p<0.0001.
